# Arbuscular mycorrhiza mitigates zinc stress on *Eucalyptus grandis* through regulating metal tolerance protein gene expression and ionome uptake

**DOI:** 10.3389/fpls.2022.1022696

**Published:** 2022-11-07

**Authors:** Li-Na Han, Si-Jia Wang, Hui Chen, Ying Ren, Xian-An Xie, Xing-Yang Wang, Wen-Tao Hu, Ming Tang

**Affiliations:** Guangdong Laboratory for Lingnan Modern Agriculture, State Key Laboratory of Conservation and Utilization of Subtropical Agro-bioresources, College of Forestry and Landscape Architecture, South China Agricultural University, Guangzhou, China

**Keywords:** *Eucalyptus grandis*, auxin biosynthesis–related genes, arbuscular mycorrhiza, metal tolerance protein, nutrient uptake, zinc stress

## Abstract

Arbuscular mycorrhizal (AM) fungi are symbionts of most terrestrial plants and enhance their adaptability in metal-contaminated soils. In this study, mycorrhized and non-mycorrhized *Eucalyptus grandis* were grown under different Zn treatments. After 6 weeks of treatment, the growing status and ionome content of plants as well as the expression patterns of metal tolerance proteins and auxin biosynthesis–related genes were measured. In this study, mycorrhized *E*. *grandis* showed higher biomass and height at a high level of Zn compared with non-mycorrhized plants. In addition, AM plants accumulated P, Mg, and Mn in roots and P, Fe, and Cu in shoots, which indicate that AM fungi facilitate the uptake of ionome nutrients to promote plant growth. In addition, mycorrhiza upregulated the expression of *EgMTP1* and *EgMTP7*, whose encoding proteins were predicted to be located at the vacuolar membrane. Meanwhile, Golgi membrane transporter *EgMTP5* was also induced in AM shoot. Our results suggest that AM likely mitigates Zn toxicity through sequestrating excess Zn into vacuolar and Golgi. Furthermore, the expression of auxin biosynthesis–related genes was facilitated by AM, and this is probably another approach for Zn tolerance.

## Introduction

Zinc (Zn) was an utmost important micronutrient for all living organisms, and it acted as catalytic and structural component in a large number of enzymes and regulatory proteins (Maret, 2009; Zhang et al., 2018; Kaur and Garg, 2021; Bae et al., 2022). Zn played an important role in regulating plant growth and development, which involves modulating a wide range of physiological processes: cell proliferation, respiration, auxin biosynthesis, and antioxidative defenses (Broadley et al., 2007; Zhang et al., 2018; Kaur and Garg, 2021; Bae et al., 2022). However, high concentration of Zn can be toxic. Excess Zn strongly decreased fresh weight and inhibited net photosynthetic rate, transpiration, and stomatal conductance in bean seedlings (Vassilev et al., 2011). In rice, a high level of Zn induced the lateral root formation through modulating the redistribution of auxin in root tips (Zhang et al., 2018) as well as inhibited the root-to-shoot translocation and distribution of P into new leaves by downregulating P transporter genes (Ding et al., 2021).

To maintain the intracellular Zn level within physiological limit, plants had developed a dynamic system involving Zn uptake, efflux, transport, and sequestration *via* particular transporters (Clemens et al., 2002; Stephens et al., 2011; Sinclair and Krämer, 2012). Zn transporters in plant included zinc/iron-regulated transporter-like proteins (ZIP), metal tolerance protein (MTP), heavy metal ATPases (HMA), natural resistance-associated macrophage protein (NRAMP), yellow stripe-like transporter family (YSL), ATP-binding cassette transporters (ABC), zinc-induced facilitator 1 proteins (ZIF1), and plant cadmium resistance proteins (PCR) (Sinclair and Krämer, 2012; Neeraja et al., 2018; Kaur and Garg, 2021). Zn toxicity resulted in suppressed expression of *ZmZIP4*, *ZmZIP5*, *ZmZIP7*, and *ZmZIP8* in shoots and *ZmZIP3* in maize roots (Li et al., 2013). In response to Zn stress, upregulation of *MsZIP2* was a detoxification mechanism to store excess Zn in xylem parenchyma cells of *Medicago sativa* (Cardini et al., 2021). Enhanced expression of *ZIF1* by excess Zn had also been verified in *Arabidopsi thaliana* (Haydon and Cobbett, 2007). Moreover, the transcript amount of *HMA4* was elevated in the roots and shoots of *M*. *sativa* exposed to surplus Zn (Cardini et al., 2021).

MTP family as divalent cation transporters involved in metal ion efflux from the cytoplasm into subcellular compartments or to extracellular space (Sinclair and Krämer, 2012) and played a pivotal role in alleviating heavy metal toxicity. Previously, MTP family had been investigated at the genomic level in *A. thaliana*, *Oryza sativa*, *Citrus sinensis*, *Populus trichocarpa*, and *Glycine max* (Gustin et al., 2011; Fu et al., 2017; Gao et al., 2020; Haque et al., 2022). According to substrate specificity, the members of MTP family were phylogenetically classified into three subfamily: Zn-CDF (to transport Zn, Cd, Ni, and Co), Zn/Fe-CDF (to transfer Fe, Zn, Cd, Ni, and Co), and Mn-CDF (mostly, to target Mn) (Montanini et al., 2007). In *A. thaliana*, five MTPs had been reported to transport Zn: AtMTP1, AtMTP2, AtMTP3, AtMTP5, and AtMTP12 (Desbrosses-Fonrouge et al., 2005; Arrivault et al., 2006; Fujiwara et al., 2015; Sinclair et al., 2018).

Arbuscular mycorrhizal (AM) fungi were obligate biotrophic fungi, which formed mutualistic symbiosis with more than 70% of terrestrial vascular plants (Brundrett and Tedersoo, 2018; Tedersoo et al., 2020). In addition, AM fungi were eco-friendly and effective in alleviating heavy metal stress of plants (Ferrol and Tamayo, 2016; Nuria et al., 2016; Tedersoo et al., 2020; Riaz et al., 2021). For example, when plants were cultivated in soils containing toxic amount of Zn, AM fungi symbiosis induced higher phosphorus (P) concentration and lower Zn concentration in shoots than those grown in control conditions (Díaz et al., 1996). In addition, mycorrhization increased the total chlorophyll content of plant grown in metal-polluted soil but diminished the concentration of H_2_O_2_ and activity of glutathione reductase (GR), catalase (CAT), guaiacol peroxidase (POD) and ascorbate peroxidase (APX) (Fernández-Fuego et al., 2017). Glomalin-related soil protein, a kind of glycoprotein produced by AM fungi, was able to combine with metal ions to sequester them in soil, consequently, to mitigate metal uptake by plants (Yang et al., 2017). AM fungi increased the resistance of host to Zn stress by upregulating the expression of ZNT:4, COPT/Ctr:2, YSL:3, and CE:1 (Wang et al., 2022).

Eucalypts was well known for its fast growth and superior hardwood, and it had been widely planted as economical tree. Furthermore, eucalypts was popular for reclamation of degraded land in coal mines, because of its ability to uptake heavy metals from contaminated soil (Maiti and Rana, 2017). Previous studies showed that *Eucalyptus grandis* can form symbiosis relationships with AM fungi in both plantation and natural woodland community, and symbiosis protected it from potential damage of heavy metals (Adams et al., 2006; Chen et al., 2007; Canton et al., 2016). With the publication of *E. grandis* genome (Myburg et al., 2014), molecular mechanisms of *E. grandis* on metals stress need further exploration.

To get further insight into the role of *E. grandis* MTP on Zn homeostasis, we analyzed their expression patterns with/without AM fungi under different Zn treatments. We also assess the effects of Zn and AM fungi on ionome content and expression of auxin biosynthesis–related genes in *E. grandis*. This study will be helpful to the development of molecular markers for cultivar breeding of *E. grandis* with a high Zn tolerance.

## Materials and methods

### Biological materials and growth conditions

*Rhizophagus irregularis* DAOM197198 was used as the mycorrhizal fungus and was propagated on *Zea mays*. After inoculation for 3 months, roots were treated with drought for another 2 months. Spores of *R*. *irregularis* were collected by modified sucrose-gradient centrifugation (Charoenpakdee et al., 2010).

The roots containing spores were broken with a blender; then, the roots were filtered through 710-, 200-, and 45-µm pore sieves. After backwashing the contents of 45-µm sieve into a 50-ml centrifuge tube, an equal volume of 50% (w/v) sucrose solution was gently added into the centrifuge tube. Then, the tubes were centrifuged at 2,000 rpm for 1 min with bench centrifuge. The spores were collected on 45-µm pore sieve and washed thoroughly to remove traces of sugar solution. Last, spores were backwashed into tube.

One milliliter of the liquid containing spores collected by sucrose-gradient centrifugation was dropped onto Miracloth (Calbiochem). The number of spores on Miracloth was counted with microscope, and the total number of spores was calculated according to the volume of the mixing liquid. Thus, we calculated the volume of liquid containing about 500 spores.

*Eucalyptus grandis* was used as host plant in this study. Seeds were surface-sterilized with 1.5% sodium hypochlorite for 15 min and washed with sterile water for three times and then were cultured in a quarter-strength Murashige and Skoog medium (pH 5.9) with 3 g L^−1^ agar. After 4 weeks, the seedlings were transferred to pots that contained sterile sands (the sands were sterilized three times for 2 h at 121°C) and inoculated with or without *R. irregularis* (about 500 spores per plant). The seedlings were cultivated in a greenhouse at 24°C/18°C day/night temperature under 16-h daylight and 50%–60% humidity. Moreover, the seedlings were fertilized with modified Long-Ashon solution (30 μM KH_2_PO_4_; Hewitt, 1966) every 3 days. After 5 weeks, mycorrhiza formation was checked following the MYCOCALC program (http://www2.dijon.inra.fr/mychintec/Mycocalc-prg/download.html). Then, the seedlings were fertilized with the abovementioned modified Long-Ashon solution containing 5, 50, and 150 μM Zn once a week for 6 weeks, respectively (Fu et al., 2017; Gao et al., 2020; Wang et al., 2021). Before harvest, fresh weight and length of root and shoot were measured. Then, roots and shoots were separated and frozen immediately in liquid nitrogen and then were stored in −80°C refrigerator.

### Elemental concentration analyses

To measure Zn, P, Mg, Fe, Cu, and Mn concentrations in *E*. *grandis*, the roots and shoots were dried in vacuum lyophilizer (Christ, Germany). After fine grounding, the samples were weighed and then were digested in 1 ml of 6 M nitric acids at 90°C for 2 h. The digested product was diluted with equal volume of sterile water and then was filtered. After a further dilution (1:10), the element concentrations were analyzed with inductively coupled plasma optical emission spectrometry (710-ES, VARIAN, USA) (Xie et al., 2021, 2022).

### Phylogenetic analyses

The MTP sequences of *A*. *thaliana*, *O*. *sativa*, *G*. *max*, *C*. *sinensis*, *P*. *trichocarpa*, and *E. grandis* were obtained from the NCBI (www.ncbi.nlm.nih.gov) and Phytozome database (phytozome-next.jgi.doe.gov) (Gustin et al., 2011; Fu et al., 2017; Gao et al., 2020; Haque et al., 2022). The sequences of identified MTP were listed in **Table S1**. The sequences were aligned with Clustal W. MEGA 7.0 was used to construct neighbor-joining tree with 1,000 bootstrap trials, and the evolutionary distance was analyzed with the Poisson correction method (Qi et al., 2022).

### Gene expression analyses

Total RNA was extracted from the roots and shoots of *E*. *grandis* based on the modified CTAB-LiCl approach (Singh et al., 2015). cDNA was synthesized from 1 μg of total RNA with HiScript III RT SuperMix for qPCR (+gDNA wiper) kit (Vazyme, Nanjing, China), and then, it was three-fold diluted with sterile water. Real-time PCR were performed using Bio-Rad iQ5 and ChamQ Universal SYBR qPCR Master Mix (Vazyme, Nanjing, China). The relative expression level of MTP in both roots and shoots of *E*. *grandis*, as well as auxin biosynthesis–related genes in roots, were normalized with the normalization factor *EgUBI3* and presented as 2^−ΔΔCt^ (Livak and Schmittgen, 2001). For the expression of *EgMTPs*, the expression level in NM roots with 5 μM Zn was defined as 1. The gene-specific primers for real-time PCR were summarized in **Table S2**.

### Mycorrhizal colonization

Fresh AM roots were fixed in 10% KOH (W/V) solution for 2 weeks under 37°C, and the solution was renewed every 3 days. After washing with sterile water, the roots were neutralized with 2% HCl (W/V) for 15 min. After another washing twice with sterile water, the roots with WGA-Alexa Fluor 488 (WGA488) then stained for 2 h at room temperature (Xie et al., 2021). Mycorrhizal colonization was quantified following the MYCOCALC program. We also launched confocal microscopy analysis performed with Zeiss 780 laser scanning confocal microscope.

### Statistical analyses

Data were analyzed by SPSS software version 19.0 (Chicago, USA). The effects of mycorrhization and Zn on gene expression and ionome concentrations were evaluated by one-way analysis of variance. Results were indicated as mean ± standard error of at least three biological replicates. Statistical differences were calculated using the Student’s *t*-test with *P* < 0.05 as the significance thresholds. In addition, GraphPad Prism (version 8.0), TBtools (Chen et al., 2020), and iTOL were used to display graphics.

## Results

### Effect of Zn and mycorrhiza on the growth of *E. grandis*

**Figure 1:**
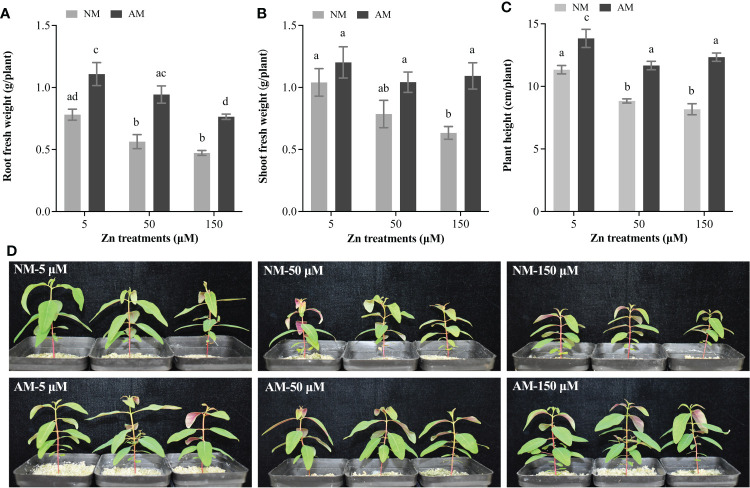
Effects of Zn and AM fungi on the growth of *E*. *grandis.* **(A)** Root fresh weight; **(B)** shoot fresh weight; **(C)** plant height; **(D)** the phenotype of *E*. *grandis* with or without AM fungi under 5, 50, and 150 μM Zn treatment. NM, non-mycorrhizal plants; AM, mycorrhizal plants. Values were indicated as mean ± SE of six biological replicates. Different letters above bars indicated significant differences at *P* < 0.05.

Regarding the effects of Zn stress on non-mycorrhizal (NM) plants, Zn treatment led to decrease of root and shoot fresh weight (**Figures 1A, B**). Inoculation with AM fungi improved both roots and shoots fresh weight of *E. grandis*, especially under 150 μM Zn treatment (**Figures 1A, B**). Considering AM plants, Zn stress on the fresh weight of roots was prominent, whereas it was not indistinctive for the shoots.

With the increase of Zn concentrations, the plant height was suppressed in NM plants, whereas AM fungi inoculation eased the suppression on the height of *E. grandis* from Zn (**Figure 1C, D**). However, mycorrhiza cannot completely eliminate the inhibition by Zn stress on plant height: The height of AM plants at 50 and 150 μM Zn was still significantly lower than at 5 μM Zn (**Figure 1C, D**).

### Effect of Zn and mycorrhiza on the ionome of *E. grandis*

**Figure 2:**
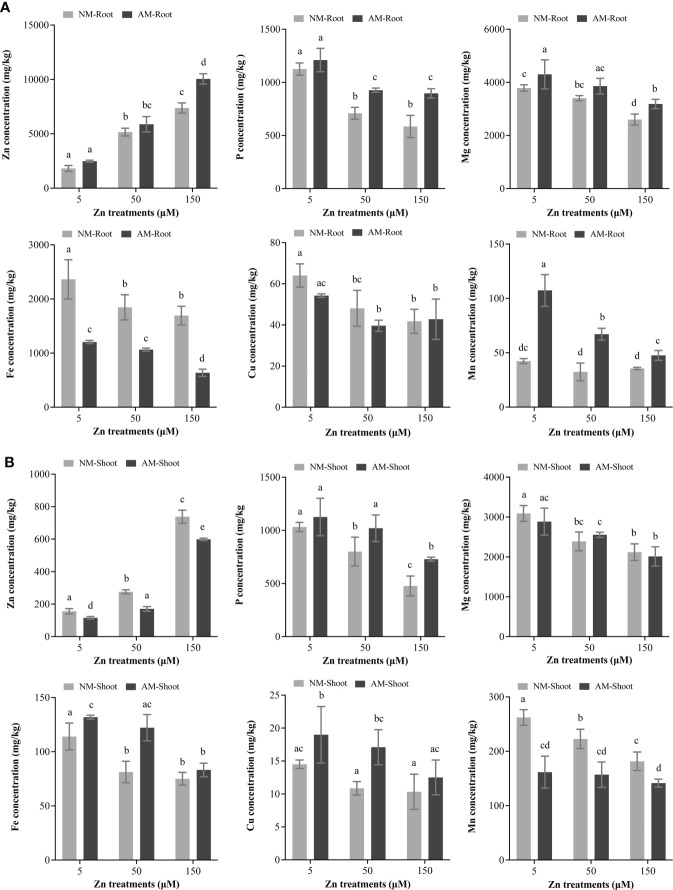
Total mineral element concentrations. The microelements (Zn, Fe, Cu, and Mn) and macroelements (P and Mg) of roots and shoots were analyzed by ICP-OES. NM, non-mycorrhizal plant; AM, mycorrhizal plant. **(A)** Total mineral concentration in root. **(B)** Total mineral concentration in shoot. Values were indicated as mean ± SE. Different letters above the bars indicated significant differences between treatments (*P* < 0.05).

Nutrient interactions in plants, in response to variable environmental stresses, significantly affect plant survival and development. With Zn treatment, Zn accumulated in NM roots, whereas the contents of P, Mg, Fe, and Cu decreased (**Figure 2A**). Meanwhile, Zn accumulation also occurred in NM shoots, but the concentrations of P, Mg, Fe, and Mn declined (**Figure 2B**). Moreover, the concentration of Mn in roots and Cu in shoots was unaffected.

Compared with NM roots, the concentration of Zn, P, Mg, and Mn was much higher in AM roots; nevertheless, Fe decreased, and Cu remained unaffected (**Figure 2A**). On the other hand, Zn and Mn contents were lower in AM shoots than that in NM shoots; P, Fe, and Cu were accumulated in AM shoots (**Figure 2B**).

### MTPs of E. grandis

**Figure 3:**
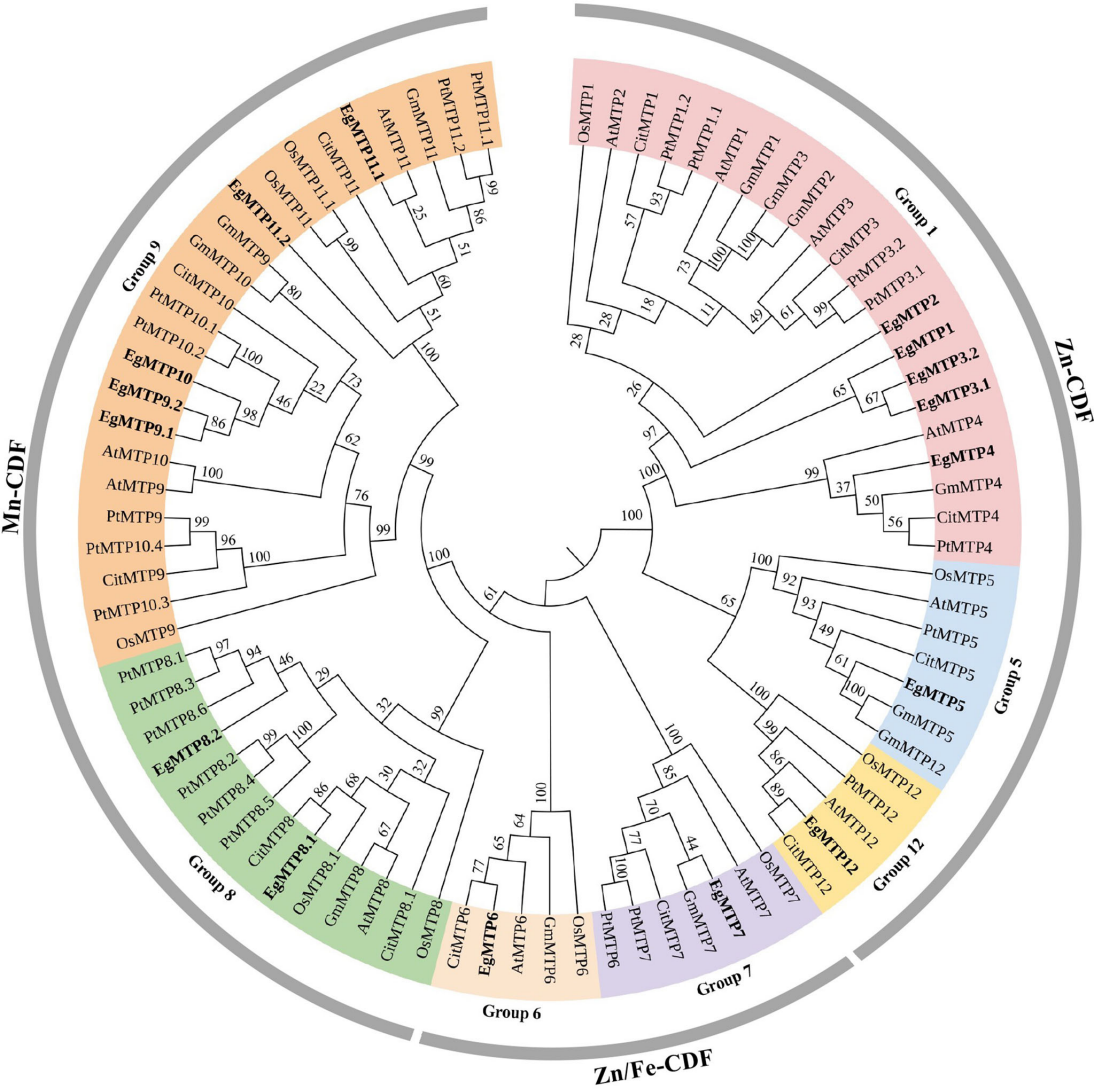
Phylogenetic relationships of MTP. The neighbor-joining tree was generated using MEGA 7.0 with 1,000 bootstrap replicates. *E. grandis* MTP proteins were in bold. At, *Arabidopsis thaliana*; Os, *Oryza sativa*; Gm, *Glycine max*; Pt, *Populus trichocarpa*; Cit, *Citrus sinensis*; Eg, *Eucalyptus grandis*.

MTP sequences of *A*. *thaliana* were used as queries to search against *E*. *grandis* genome in Phytozome database to identify the MTP genes of *E*. *grandis*. After the conserved domain analysis, a total number of 16 MTP encoding genes were identified: *EgMTP1*, *EgMTP2*, *EgMTP3*.*1*, *EgMTP3*.*2*, *EgMTP4*, *EgMTP5*, *EgMTP6*, *EgMTP7*, *EgMTP8*.*1*, *EgMTP8*.*2*, *EgMTP9*.*1*, *EgMTP9*.*2*, *EgMTP10*, *EgMTP11*.*1*, *EgMTP11*.*2*, and *EgMTP12* (**Figure 3**).

To better understand the evolutionary characteristics and possible functions of the MTP family in *E*. *grandis*, a neighbor-joining tree consisting of 84 MTPs (including 12 MTPs from *A*. *thaliana*, 10 from *O*. *sativa*, 12 from *G*. *max*, 12 from *C*. *sativus*, 22 from *P*. *trichocarpa*, and 16 from *E*. *grandis*) was constructed (**Figure 3**). As previously reported, these proteins were divided into Zn-CDF, Zn/Fe-CDF, and Mn-CDF clusters (Montanini et al., 2007; Gustin et al., 2011), with seven (EgMTP1 to EgMTP5 and EgMTP12), two (EgMTP6 and EgMTP7), and seven (EgMTP8.1 to EgMTP11.2) MTP members, respectively (**Figure 3**).

### Effect of Zn and mycorrhiza on the expression of *EgMTPs*

Previously, the expression of *ZIP2* was upregulated by excess Zn and downregulated by AM symbiosis both in *Medicago truncatula* and *Astragalus sinicus* (Burleigh et al., 2003; Xie et al., 2021). Herein, we analyzed the expression patterns of MTPs in NM and AM plants supplied with different concentrations of Zn.

**Figure 4:**
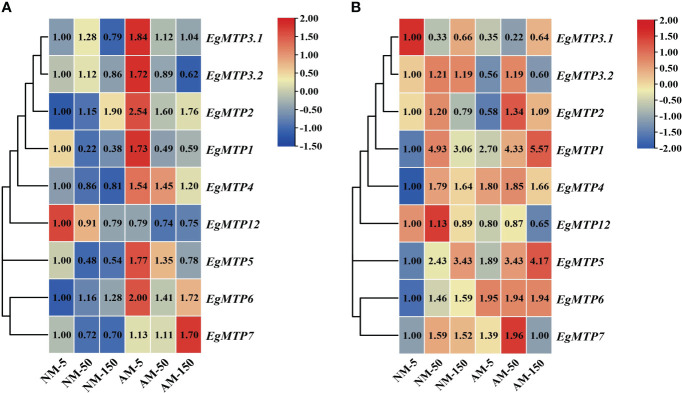
Expression profiles of *EgMTP*. The heat map was generated using the expression fold changes of *EgMTP* family. Relative gene expression was calculated by the 2^−ΔΔCT^ method using the *EgUBI3* as a normalizer. For each gene, the expression level in NM roots with 5 μM Zn was defined as 1. **(A)** The expression profiles of *EgMTPs* in root. **(B)** The expression profiles of *EgMTPs* in shoot.

In NM roots, the expression of *EgMTP1*, *EgMTP5*, and *EgMTP7* were significantly repressed by high-Zn treatment, whereas *EgMTP2* presented an overall upward trend, and no significant difference was detected in *EgMTP3.1*, *EgMTP3.2*, *EgMTP4*, *EgMTP6*, and *EgMTP12* (**Figure 4A**). As for AM root, Zn mostly reduced the expression of *EgMTPs*, except for *EgMTP7*. However, compared with NM roots, mycorrhiza induced the expression of *EgMTP1*, *EgMTP5*, and *EgMTP7* (**Figure 4A**). Regarding to NM shoots, the expression pattern of *EgMTP3.1* showed a downward trend with increased Zn concentration, whereas *EgMTP1*, *EgMTP4*, *EgMTP5*, *EgMTP6*, and *EgMTP7* were upregulated (**Figure 4B**). In AM shoots, Zn treatment induced the expression of *EgMTP1*, *EgMTP2*, *EgMTP3.1*, and *EgMTP5*, and upregulated *EgMTP1* and *EgMTP5* compared with that in NM shoots (**Figure 4B**). Overall, mycorrhiza induced the expression of *EgMTP1*, *EgMTP5*, and *EgMTP7* in roots and *EgMTP1* and *EgMTP5* in shoots.

### Effect of Zn and mycorrhiza on the expression of the auxin biosynthesis–related gene

**Figure 5:**
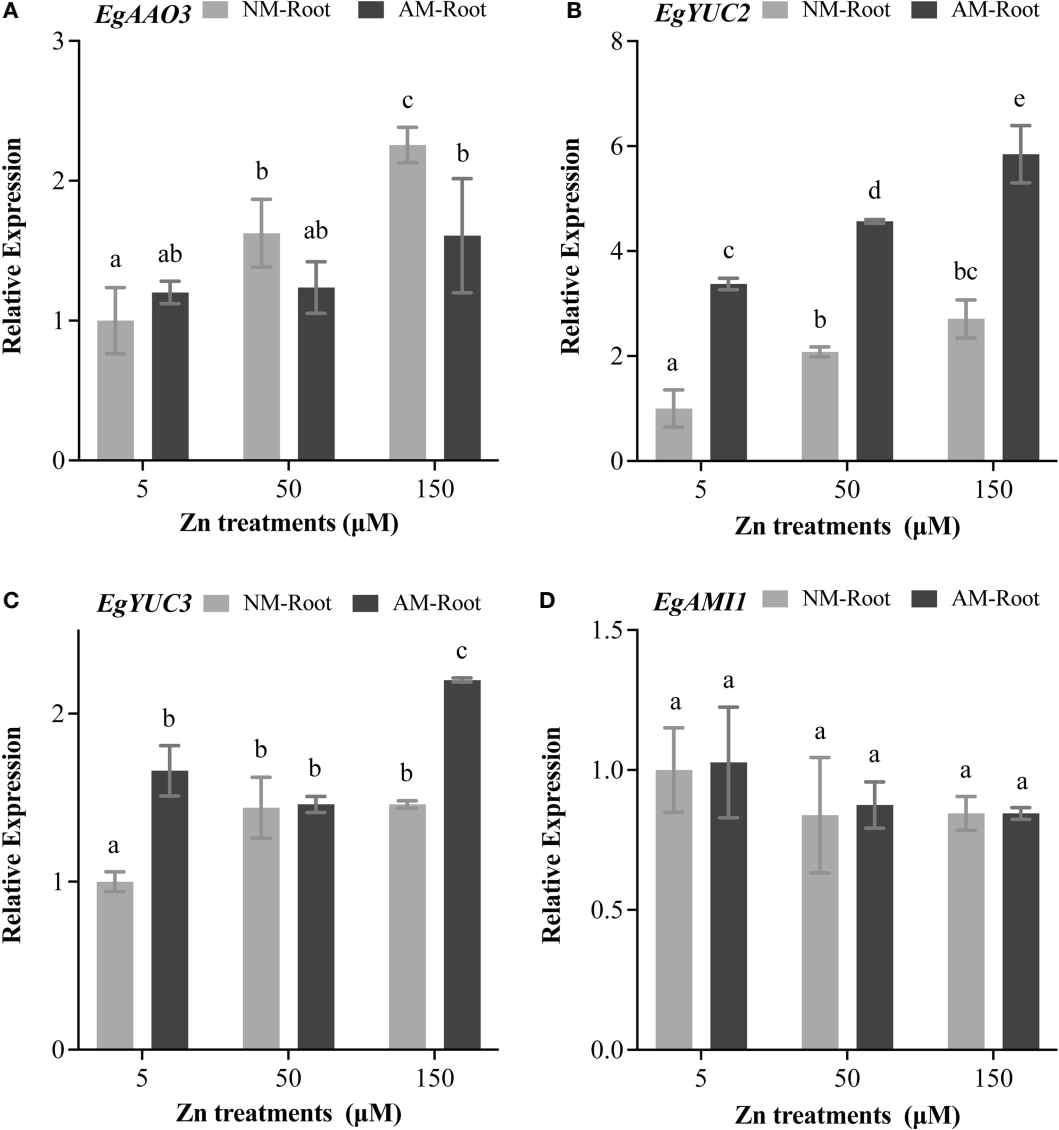
Expression profiles of auxin biosynthesis–related genes. The relative expression of *EgAAO3* **(A)**, *EgYUC2* **(B)**, *EgYUC3* **(C)**, and *EgAMI1* **(D)** was calculated by the 2^−ΔΔCT^ method using the *EgUBI3* as a normalizer. For each gene, the expression level in NM roots with 5 μM Zn was defined as 1. NM-Root, the root of non-mycorrhizal plants; AM-Root, the root of mycorrhizal plants. Values were indicated as mean ± SE. Different letters on the histograms indicated significant differences (*P* < 0.05).

To further investigate the effects of Zn stress on auxin biosynthesis in *E*. *grandis*, we analyzed the expression patterns of *EgAAO3*, *EgYUC2*, *EgYUC3*, and *EgAMI1*. *EgAAO3* was strongly induced at 50 and 150 μM Zn (**Figure 5A**). Meanwhile, *EgYUC2* and *EgYUC3* significantly expressed at 150 μM Zn (**Figures 5B, C**), and the expression of *EgAMI1* was unaffected under any Zn concentration (**Figure 5D**).

Chareesri et al. (2020) and Wang et al.(2021) demonstrated that, compared to the NM roots, the content of auxin in the mycorrhizal rice and tomato roots significantly increased. To elucidate how mycorrhiza increased auxin accumulation in AM roots, we evaluated the transcript level of *EgAAO3*, *EgYUC2*, *EgYUC3*, and *EgAMI1*. Compared with NM roots, the expression of *EgYUC2* and *EgYUC3* was induced in AM roots (**Figures 5B, C**), whereas *EgAAO3* was repressed at 150 μM Zn treatment (**Figure 5A**).

### Mycorrhizal colonization

**Figure 6:**
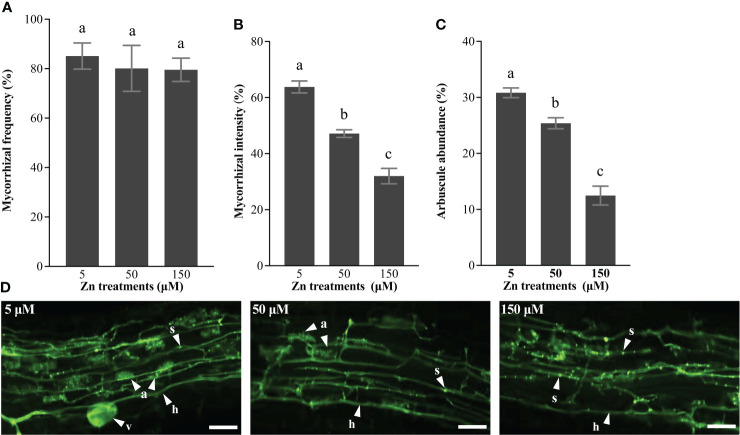
Mycorrhizal colonization. **(A)** Mycorrhizal frequency, **(B)** mycorrhizal intensity, and **(C)** arbuscule abundance were quantified using the MYCOCALC program. Values were indicated as mean ± SE. Different letters on the histograms indicated that the means significant differences (*P* < 0.05). **(D)** a, arbuscule; h, hyphae; v, vesicles; s, septa; bar, 50 μm.

To analyze the effect of Zn stress on AM fungi development in *E*. *grandis*, we quantified the mycorrhizal colonization rates in roots inoculated with *R*. *irregularis*. Plants grown with 5, 50, and 150 μM Zn showed similar mycorrhizal frequency (**Figure 6A**), based on the percentage of roots colonized by AM fungi in the whole roots. Although symbiosis had already existed for 5 weeks before the Zn treatment, mycorrhizal intensity was lower in AM roots grown at 50 and 150 μM Zn compared with that at 5 μM Zn (**Figure 6B**). Moreover, the arbuscule numbers decreased in AM roots exposed to a high level of Zn (**Figure 6C**). Roots exposed to 5 μM Zn had normal arbuscules, with full hyphal branches in the cortical cells, but fewer arbuscules were formed with high-Zn treatment (**Figure 6D**). In addition, intraradical hyphae at 150 μM Zn contained more septa (**Figure 6D**), which was a morphological signature of degradation of AM fungi. Intraradical hyphae was the channel for transporting nutrients in AM fungi, and the formation of septa prevented transport of nutrients to arbuscule, which led to the death of the arbuscule. In a nutshell, high-Zn treatments disturbed the arbuscule development through forming more septa in intraradical hyphae.

## Discussion

### Mycorrhiza promotes the growth of *E. grandis* under Zn stress

AM fungi were beneficial symbionts of plants that increased host resistance to various environmental stresses. Mycorrhizal *Betula pubescens* had higher fresh and dry weight than NM plants in metal-polluted industrial soil (Fernández-Fuego et al., 2017). In Cu-contaminated soils, symbiosis facilitated Cu tolerance of maize with increasing fresh weight (Gómez-Gallego et al., 2022). In our study, significantly higher biomass and height were detected in mycorrhizal *E*. *grandis* (**Figure 1**). AM fungi absorbed nutrients beyond the depletion zone that develops around the roots through external mycelium and then delivers them to host roots (Wang et al., 2017; Xie et al., 2021; Gómez-Gallego et al., 2022). This was a quite effective way to promote plant growth, and higher biomass further enhances the Zn tolerance of plants.

### Zn and mycorrhiza affect the uptake of mineral elements

The growth and development of higher plants needed at least 17 essential elements, among which P and Mg were known as macroelements; Fe, Zn, Cu, and Mn were regarded as microelements; and the interactions between macro- and microelement were one of the key processes in the life cycle of plants (Xie et al., 2019; Fan et al., 2021; Kumar et al., 2021). Previous studies indicated that excess Zn triggered P starvation in lettuce and rice (Bouain et al., 2014; Ding et al., 2021). Similarly, we observed Zn increasing and P decreasing in both root and shoot of *E. grandis* along with elevation of Zn treatment (**Figure 2**). High Zn downregulated the expression of uptake and transporter-related genes of P (Ding et al., 2021), which inhibited the uptake and translocation of P; thus, less P was transported to shoots under high-Zn treatment (**Figure 2**). Consisting with the case of rapeseed seedlings (Wang et al., 2009), the concentrations of Fe and Mg in roots and shoots of *E. grandis* decreased with surplus Zn (**Figure 2**). On the other hand, when maize was grown in high-Zn condition, the concentrations of K, Ca, Mg, Fe, Mn, Ni, and Co significantly decreased in root, and Mn and Cu diminished in shoot along with the increase of S, Mg, and Mo (Bokor et al., 2015). Excessive Zn affected the level of Fe sensing, resulting in Fe deficiency (Leskova et al., 2017). However, Cu content reduced in roots and Mn increased in shoots of *E. grandis* with a high level of Zn (**Figure 2**). Cross-talks between mineral nutrients involved with complicated mechanisms; therefore, multi-level interactions among nutrient elements needed further explore to better understand their availability.

AM fungi can improve shoot biomass and retain metals in roots to restrict their translocation to aerial parts under heavy metal stress (Huang et al., 2018; Janeeshma and Puthur, 2020; Riaz et al., 2021). In our study, more Zn was accumulated in AM roots; less Zn, therefore, was transferred to AM shoots (**Figure 2**). Moreover, AM fungal hyphal network functionally extended the root system of hosts, granting the hosts the ability to uptake mineral nutrients from enlarged soil volume to enhance the metal tolerance of hosts (Göhre and Paszkowski, 2006; Wang et al., 2017; Gómez-Gallego et al., 2022). For example, *Thlaspi praecox* grew in soils highly contaminated by Cd, Zn, and Pb, the concentrations of P, S, Ni, and Cu in both AM shoots and roots were found to be increased (Vogel-Mikuš et al., 2006). AM fungi improved the nutritional (P, N, Mg, and Fe) and water status, and stimulated proline biosynthesis of hosts, which enhanced the tolerance to Cd and Zn (Garg and Singh, 2018). In addition, concentrations of P, K, Mg, and Ca in mycorrhizal maize grown in Cu-contaminated soil were often higher than that in NM plants (Gómez-Gallego et al., 2022). Mycorrhiza significantly increased the concentrations of P, Mg, and Mn in *E. grandis* roots, as well as P, Fe, and Cu in shoots. However, Fe concentration in AM roots was lower than that in NM roots but higher in AM shoots than that in NM shoots (**Figure 2**), probably because the effect of Zn accumulation was dominant and stimulates Fe transporting from root to shoot.

To sum up, high Zn threatens plant growth by disturbing the homeostasis of nutrient elements, and AM fungi eases the stress to some extent.

### Zn and mycorrhiza regulate the expression of *EgMTPs*

MTPs played vital roles in exporting excess metal ions into subcellular compartments or to extracellular space (Desbrosses-Fonrouge et al., 2005; Arrivault et al., 2006; Fujiwara et al., 2015; Migocka et al., 2018; Sinclair et al., 2018; Gao et al., 2020). Because MTPs of Mn-CDF cluster mostly transported Mn (Montanini et al., 2007), we analyzed the expression patterns of MTPs of Zn-CDF and Zn/Fe-CDF clusters. As showed in **Figure 3**, EgMTP1, EgMTP2, EgMTP3.1, EgMTP3.2, EgMTP4, EgMTP5, EgMTP12, and EgMTP7 of *E*. *grandis* had high similarity with MTPs of *A*. *thaliana*, *G. max*, *P. trichocarpa*, and *C*. *sinensis*, which implied that they shared comparable functions. Previous studies reported that AtMTP1 and AtMTP3 were vacuolar membrane transporters (Desbrosses-Fonrouge et al., 2005; Arrivault et al., 2006), and MTP1, MTP3, MTP4, and MTP7 of *G. max*, *P. trichocarpa*, and *C. sinensis* were also predicted to be located at vacuole (Fu et al., 2017; Gao et al., 2020; Haque et al., 2022). AtMTP5, AtMTP12, and CsMTP5 were localized at the Golgi compartment (Fujiwara et al., 2015; Migocka et al., 2018), whereas AtMTP2 and EgMTP6 were endoplasmic reticulum membrane proteins (Sinclair et al., 2018; Han et al., 2022). According to the grouping in the tree (**Figure 3**), we thus assumed that EgMTP1, EgMTP3.1, EgMTP3.2, EgMTP4, and EgMTP7 were located at vacuole; EgMTP2 and EgMTP6 were localized in the endoplasmic reticulum membrane; and EgMTP5 and EgMTP12 were membrane transporters of Golgi apparatus.

Desbrosses-Fonrouge et al. (2005) found that AtMTP1 acted to exclude excess Zn into vacuoles and driven Zn accumulation in young leaves. In this study, the expression of *EgMTP1* was suppressed in root but induced in NM shoot under Zn treatment (**Figure 4**); the expression pattern was similar with that of *PtrMTP1* (Gao et al., 2020). As Zn can enter plant cell non-specifically through plasma membrane transport proteins (Arrivault et al., 2006), the overaccumulated Zn in root suppressed the expression of *EgMTP1* and enhanced the transfer of Zn from root to shoot; then, excessive Zn in shoot upregulated the expression of *EgMTP1* to promote the storage of Zn in shoot vacuole. AtMTP3 mostly expressed in root and functioned in the immobilization of Zn in root vacuoles, restricting the movement of Zn from root into shoot (Arrivault et al., 2006). The expression of *EgMTP3.1* in NM shoot was reduced under Zn oversupply, indicating that EgMTP3.1 mediated Zn exclusion from shoot. Conversely, *EgMTP4* in NM shoot was induced under Zn oversupply (**Figure 4**). We speculated that EgMTP4 and MTP3 had difference physiological functions, and EgMTP4 promoted the Zn storage in shoot vacuoles. The expression level of *EgMTP7* was strongly intensified by Zn in NM shoot (**Figure 4**), a case similar with *PtMTP7* (Gao et al., 2020), suggesting that EgMTP7 transported Zn into shoot vacuole to remit the Zn toxicity.

AtMTP5 and AtMTP12 formed functional heterodimer to load Zn into Golgi, but the expression of *AtMTP12* was irrelevant to Zn concentration (Fujiwara et al., 2015). Similarly, the expression of *EgMTP12* in NM plants was not affected by Zn (**Figure 4**). In cucumber, CsMTP5 and CsMTP12 also functioned as a heterodimeric complex, which involved in transporting Zn into Golgi compartment, and the expression of *CsMTP5* was obviously upregulated with low-Zn treatment (Migocka et al., 2018). Conversely, a high level of Zn increased the expression of *EgMTP5* in both NM roots and shoots (**Figure 4**). Therefore, we propose that the heterodimeric complex EgMTP5-EgMTP12 functions to deliver excess Zn to Golgi compartment and is regulated by zinc at the level of *EgMTP5* transcription.

AtMTP2 contributed to the root-to-shoot Zn translocation through plasmodesmus (Sinclair et al., 2018). The high transcriptional level of *EgMTP2* in NM roots responded to excess Zn (**Figure 4**); therefore, more Zn was transferred to the shoot through symplast pathway. In addition, our previous study found that the expression of *EgMTP6* was irrelevant to Zn concentration, and heterologous expression of EgMTP6 in *zrc1*-mutant yeast enhanced the Zn tolerance of *zrc1Δ*, which cannot grow in high-Zn condition (Han et al., 2022). Thus, EgMTP6 mediated the sequestration of Zn to endoplasmic reticulum in the non-transcript level.

Recently, Gómez-Gallego et al. (2022) found an enhanced expression of the vacuolar membrane transporters *ZmHMA3a* and *ZmHMA4* in AM plants under Cu stress. Mycorrhiza promoted sequestering Cu into vacuole of root and shoot to reduce Cu translocation to aerial part by regulating the genes of Cu transporters (Gómez-Gallego et al., 2022). In *Astragalus sinicus*, mycorrhiza downregulated the expression of *AsZIP2* to reduce absorbing excessive Zn (Xie et al., 2021). It seems that mycorrhiza downregulates genes involved in metal uptake and upregulates genes related to exportation to protect host plant from metals toxicity. With Zn oversupply, mycorrhiza increased the expression of *EgMTP1* and *EgMTP7* in roots, as well as *EgMTP1* and *EgMTP5* in shoots, to facilitate the transport of excess Zn into vacuole and Golgi for plant detoxifying.

### Zn and mycorrhiza affect the expression of auxin biosynthesis–related genes

In recent years, the alterations of auxin biosynthesis and transport induced by heavy metals stimuli had been intensively explored (Jiang et al., 2018; López-Ruiz et al., 2020; Angulo-Bejarano et al., 2021; Wang et al., 2021). For instance, toxic Cu, Al, Fe, and Ni disturbed auxin biosynthesis and distribution in root tips to inhibit root growth and development (Wu et al., 2014; Li et al., 2015; Song et al., 2017; Leškovï et al., 2020). Different from excess Se that decreased the auxin biosynthesis *via* reducing the expression of *YUCCA1* and *YUCCA3* in rice plants (Malheiros et al., 2019), excess Zn enhanced the expression of *EgAAO3*, *EgYUC2*, and *EgYUC3* (**Figure 5**). Previously, a comparative transcriptomic investigation indicated that 24-h treatment of 200 μM Zn significantly induced the expression of auxin biosynthesis genes (ATP SULFURYLASE ARABIDOPSIS1, SUPERROOT1, TRYPTOPHAN AMINOTRANSFERASE OF ARABIDOPSIS1, YUC2, YUC3, CYTOCHROME P450, and AAO3) (Zhang et al., 2018). On the other hand, Cu toxicity was found to inhibit auxin biosynthesis *via* reducing the expression of *TAA1* and *YUCCA* (Song et al., 2017). In contrast, transcriptomic analyses of auxin biosynthetic-related genes, including auxin amide synthase and tryptophan synthase, showed that Cu induced their expression (Zhao et al., 2009). The discrepancies in results might be related to differences in experimental exposure time and treatment approach. However, excess Zn promoted the expression of *AAO3*, *YUC2*, and *YUC3* in *E*. *grandis*.

AM fungi had a positive effect on the regulation of the auxin levels in plants under salt stress, drought, and biotic stress, and auxin concentration in mycorrhizal plants was higher than nonmycorrhizal plants (He et al., 2017; Liu et al., 2016; Chareesri et al., 2020). Moreover, the activity of synthetic auxin-inducible promoter DR5 increased in roots colonized by *R*. *irregularis*, mainly in cells containing arbuscules (Etemadi et al., 2014). Mycorrhiza is thus likely to promote auxin synthesis. Our results showed that *EgYUC2* and *EgYUC3* were upregulated in AM roots (**Figure 5**). Furthermore, the positive correlation between auxin content and arbuscule abundance suggested that maintaining cellular auxin homoeostasis was involved in finely tuning AM symbiosis (Hanlon and Coenen, 2011). The auxin content with denser AM fungi colonization was higher than those with sparser colonization in rice (Chareesri et al., 2020). According to this study, the arbuscule number at 150 μM Zn was significantly lower than at 5 μM and 50 μM Zn, but the expression of *EgYUC2* and *EgYUC3* was higher. The result reveals that, although Zn restrains the growth of mycorrhiza, it remarkably promotes the expression of auxin synthesis genes.

## Conclusion

**Figure 7:**
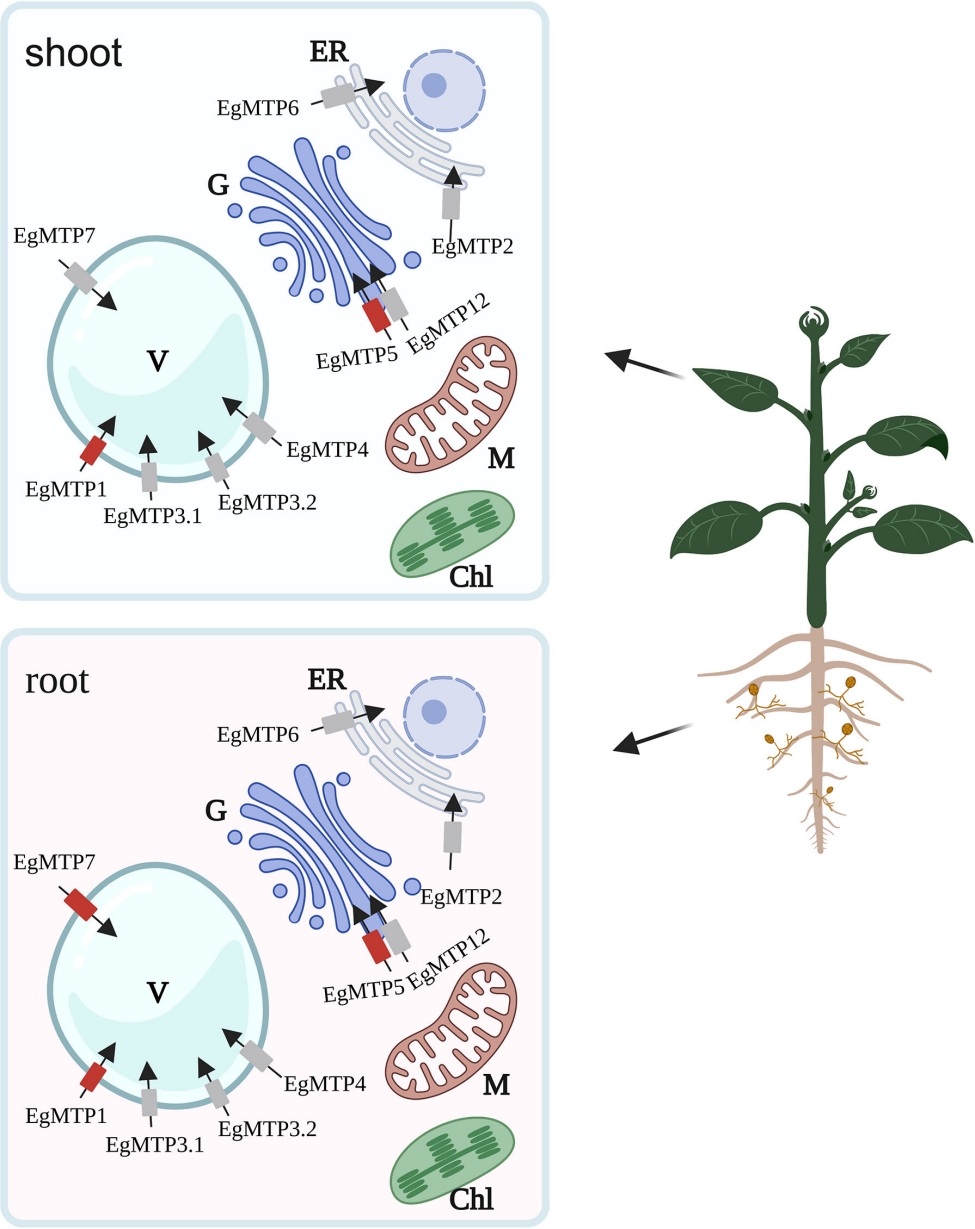
Schematic representation of the major changes induced by Zn in *EgMTP* expression in mycorrhizal and *E. grandis*. Red transporters, upregulated *EgMTPs* by mycorrhizal; ER, endoplasmic reticulum; V, vacuole; G, golgi; Chl, chloroplast; M, mitochondrion.

To sum up, we herein propose a mechanism of Zn detoxification in *E. grandis* (**Figure 7**): EgMTP1, EgMTP3.1, EgMTP3.2, EgMTP4, and EgMTP7 involve in sequestering Zn in vacuole; EgMTP2 and EgMTP6 mediate the Zn transport into endoplasmic reticulum; and EgMTP5 and EgMTP12 load Zn into Golgi. AM fungi inoculation enhances the expression of two putative tonoplast transporters (EgMTP1 and EgMTP7) and one Golgi transporter (EgMTP5) in *E. grandis* under Zn toxicity, indicating that mycorrhiza facilitates the transfer of Zn into vacuole and Golgi. In addition, mycorrhiza promotes mineral nutrient uptake to improve the growth of *E. grandis* and induces the expression of auxin biosynthesis–related genes to improve mycorrhizal colonization to enhance the Zn tolerance. The results will be valuable to the development of molecular markers for cultivar breeding of eucalyptus with a high Zn tolerance. Further functional investigations were required to better understand their role of EgMTPs to alleviate Zn toxicity.

## Data availability statement

The raw data supporting the conclusions of this article will be made available by the authors, without undue reservation.

## Author contributions

L-NH: Conceptualization, methodology, data analysis and curation, visualization, writing, and editing. S-JW, YR, and X-YW: Methodology and data analysis. X-AX and W-TH: Methodology. HC and MT: Conceptualization, editing, and funding acquisition. All authors contributed to the article and approved the submitted version.

## Funding

This work was supported by the Key Projects of Guangzhou of Science and Technology Plan (grant no. 201904020022), the Laboratory of Lingnan Modern Agriculture Project (grant no. NZ2021025), and the National Natural Science Foundation of China (grant nos. 31800092 and 32071639).

## Acknowledgments

We particularly thank Lei Duan of South China Botanical Garden, Chinese Academy of Sciences for refining the text.

## Conflict of interest

The authors declare that the research was conducted in the absence of any commercial or financial relationships that could be construed as a potential conflict of interest.

## Publisher’s note

All claims expressed in this article are solely those of the authors and do not necessarily represent those of their affiliated organizations, or those of the publisher, the editors and the reviewers. Any product that may be evaluated in this article, or claim that may be made by its manufacturer, is not guaranteed or endorsed by the publisher.
