# Supplementary material for: Arbuscular mycorrhiza mitigates zinc stress on Eucalyptus grandis through regulating metal tolerance protein gene expression and ionome uptake
Source: Front Plant Sci. 2022 Nov 7;13:1022696. doi: 10.3389/fpls.2022.1022696 (PMC9676645; doi:10.3389/fpls.2022.1022696)
Supplement: Supplementary file 1 [file DataSheet_1.docx]

**Table S1.** List of MTP members used in this study.

| **Gene** | **Definition** | **Gene** | **Definition** |
| --- | --- | --- | --- |
| AtMTP1 | At2g46800 | PtMTP11.2 | Potri.008G049600 |
| AtMTP2 | At3g61940 | PtMTP12 | Potri.005G110300 |
| AtMTP3 | At3g58810 | GmMTP1 | NM_001255953.3 |
| AtMTP4 | At2g29410 | GmMTP2 | XM_014768429.3 |
| AtMTP5 | At3g12100 | GmMTP3 | NM_001255953.3 |
| AtMTP6 | At2g47830 | GmMTP4 | XM_003539492.5 |
| AtMTP7 | At1g51610 | GmMTP5 | XM_003554165.5 |
| AtMTP8 | At3g58060 | GmMTP6 | XM_003536181.5 |
| AtMTP9 | At1g79520 | GmMTP7 | NM_001252720.1 |
| AtMTP10 | At1g16310 | GmMTP8 | XM_003544292.4 |
| AtMTP11 | At2g39450 | GmMTP9 | XM_006587093.4 |
| AtMTP12 | At2g04620 | GmMTP10 | XM_003546775.5 |
| OsMTP1 | Os05g03780 | GmMTP11 | XM_003552623.5 |
| OsMTP5 | Os02g58580 | GmMTP12 | XM_003554165.5 |
| OsMTP6 | Os03g22550 | Cit MTP1 | orange1.1t00574.1 |
| OsMTP7 | Os04g23180 | Cit MTP3 | Cs5g01220.1 |
| OsMTP8 | Os02g53490 | Cit MTP4 | Cs8g19230.1 |
| OsMTP8.1 | Os03g12530 | Cit MTP5 | orange1.1t02233.1 |
| OsMTP9 | Os01g03914 | Cit MTP6 | Cs5g32700.1 |
| OsMTP11 | Os01g62070 | Cit MTP7 | Cs1g10560.1 |
| OsMTP11.1 | Os05g38670 | Cit MTP8 | orange1.1t02212.1 |
| OsMTP12 | Os08g32650 | Cit MTP8.1 | orange1.1t02053.1 |
| PtMTP1.1 | Potri.014G106200 | Cit MTP9 | orange1.1t03266.1 |
| PtMTP1.2 | Potri.002G180100 | Cit MTP10 | orange1.1t03267.1 |
| PtMTP3.1 | Potri.011G150600 | Cit MTP11 | Cs7g28390.1 |
| PtMTP3.2 | Potri.001G450900 | Cit MTP12 | Cs2g28860.1 |
| PtMTP4 | Potri.001G245800 | EgMTP1 | Eucgr.D01644.1 |
| PtMTP5 | Potri.016G045200 | EgMTP2 | Eucgr.E01090.1 |
| PtMTP6 | Potri.T034500 | EgMTP3.1 | Eucgr.C02043.1 |
| PtMTP7 | Potri.010G251300 | EgMTP3.2 | Eucgr.D01642.1 |
| PtMTP8.1 | Potri.003G215600 | EgMTP4 | Eucgr.B02300.1 |
| PtMTP8.2 | Potri.001G010200 | EgMTP5 | Eucgr.A02454.1 |
| PtMTP8.3 | Potri.001G010300 | EgMTP6 | Eucgr.K03452.1 |
| PtMTP8.4 | Potri.001G010300 | EgMTP7 | Eucgr.F04020.1 |
| PtMTP8.5 | Potri.001G010300 | EgMTP8.1 | Eucgr.K01853.1 |
| PtMTP8.6 | Potri.001G009900 | EgMTP8.2 | Eucgr.B03634.1 |
| PtMTP9 | Potri.008G083600 | EgMTP9.1 | Eucgr.F04468.1 |
| PtMTP10.1 | Potri.010G172800 | EgMTP9.2 | Eucgr.F04469.1 |
| PtMTP10.2 | Potri.010G172900 | EgMTP10 | Eucgr.F04467.1 |
| PtMTP10.3 | Potri.010G172700 | EgMTP11.1 | Eucgr.G02893.1 |
| PtMTP10.4 | Potri.010G172600 | EgMTP11.2 | Eucgr.J01168.1 |
| PtMTP11.1 | Potri.010G211300 | EgMTP12 | Eucgr.J01747.1 |

**Table S2.** Primers used in this study.

| **Primer ID** | **Sequence (5'-3')** |
| --- | --- |
| EgMTP1Fq | TGTGCTGGGCACTACAATAAG |
| EgMTP1Rq | CTGCAGTCACATCATCCATCTC |
| EgMTP2Fq | GGAGAGTACGCCTAGAGAGATT |
| EgMTP2Rq | TAGCACCTTTCCAACCGTTATAG |
| EgMTP3.1Fq | TCTCGGTGATTTCCTCCAAAC |
| EgMTP3.1Rq | TATACGAGAGTCCCAGCAAGA |
| EgMTP3.2Fq | TCATGGCTGTAGAGGTTGTTG |
| EgMTP3.2Rq | GAAGCCCACAGAGAGAATAAGG |
| EgMTP4Fq | TCTAGTTGGAAGGCAACATCAC |
| EgMTP4Rq | GACCAGAAACCAGCCAGATAAG |
| EgMTP5Fq | TTTCGGTGGTGTCCTTCTTC |
| EgMTP5Rq | AAGAGGCCGATAGCCAATTC |
| EgMTP6Fq | TGGTTGCTCTTCTTGGTGTAG |
| EgMTP6Rq | CCAGTTTCAAGTCCAGCCTTA |
| EgMTP7Fq | CTTTAGTGATCGGAGGCTCTTT |
| EgMTP7Rq | GATCATGACCACGCCAGATAA |
| EgMTP12Fq | GCTCACGAACGGGATCTATTTA |
| EgMTP12Rq | CGTTCCCACTACCTCTGTATTC |
| EgUBI3Fq | TCACCTACGTCTACCAGAAGG |
| EgUBI3Rq | TCCTCGAAAGCTGTAAACATGG |
| EgAAO3Fq | AGTGCAACTCGATATTCCAGC |
| EgAAO3Rq | TCCTTCCCTAATCTCCAGTCC |
| EgYUC2Fq | ACCTCCCGAAGCAATTCTG |
| EgYUC2Rq | TGATTGAAAACCGGCCTCAG |
| EgYUC3Fq | TCTATGCGGTTGGGTTCAC |
| EgYUC3Rq | CCTCTGCTTCATATCCTCTTTCC |
| EgAMI1Fq | TCTTTGGCAGCTCTTTCTAGTG |
| EgAMI1Rq | CCTGACCCGTTCTGATATTCC |
